# The endocannabinoid anandamide causes endothelium-dependent vasorelaxation in human mesenteric arteries

**DOI:** 10.1016/j.phrs.2016.08.028

**Published:** 2016-11

**Authors:** Christopher P. Stanley, William H. Hind, Christina Tufarelli, Saoirse E. O’Sullivan

**Affiliations:** School of Medicine, University of Nottingham, Royal Derby Hospital, Derby, DE22 3DT, UK

**Keywords:** Cannabinoid, Anandamide, Vasorelaxation, Human, Mesenteric artery, CB_1_, Endothelium, Obesity, Paracetamol

## Abstract

The endocannabinoid anandamide (AEA) causes vasorelaxation in animal studies. Although circulating AEA levels are increased in many pathologies, little is known about its vascular effects in humans. The aim of this work was to characterise the effects of AEA in human arteries. Ethical approval was granted to obtain mesenteric arteries from patients (n = 31) undergoing bowel resection. Wire myography was used to probe the effects and mechanisms of action of AEA. RT‐PCR was used to confirm the presence of receptor mRNA in human aortic endothelial cells (HAECs) and intracellular signalling proteins were measured using multiplex technology. AEA caused vasorelaxation of precontracted human mesenteric arteries with an R_max_ of ∼30%. A synthetic CB_1_ agonist (CP55940) caused greater vasorelaxation (R_max_ ∼60%) while a CB_2_ receptor agonist (HU308) had no effect on vascular tone. AEA-induced vasorelaxation was inhibited by removing the endothelium, inhibition of nitric oxide (NO) synthase, antagonising the CB_1_ receptor and antagonising the proposed novel endothelial cannabinoid receptor (CB_e_). AEA‐induced vasorelaxation was not affected by CB_2_ antagonism, by depleting sensory neurotransmitters, or inhibiting cyclooxygenase activity. RT‐PCR showed CB_1_ but not CB_2_ receptors were present in HAECs, and AEA and CP55940 had similar profiles in HAECs (increased phosphorylation of JNK, NFκB, ERK, Akt, p70s6K, STAT3 and STAT5). Post hoc analysis of the data set showed that overweight patients and those taking paracetamol had reduced vasorelaxant responses to AEA. These data show that AEA causes moderate endothelium-dependent, NO-dependent vasorelaxation in human mesenteric arteries via activation of CB_1_ receptors.

## Graphical abstract

**Figure:**
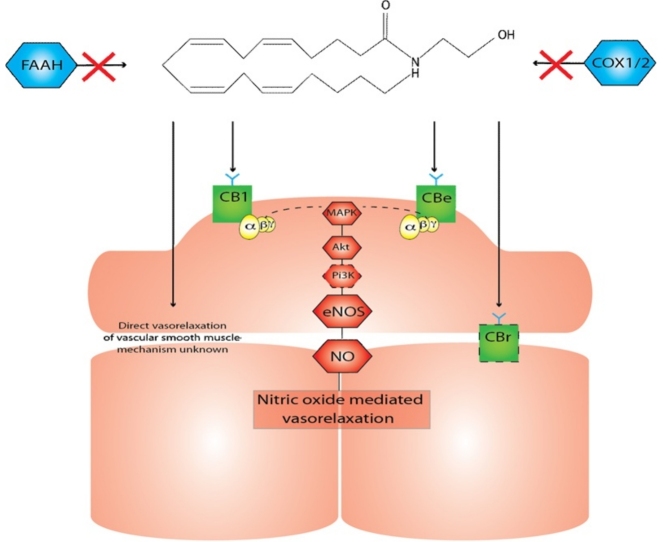

FAAH and COX1/2 do not modulate AEA-induced vasorelaxation. AEA induced-vasorelaxation is partially dependant on the CB_1_ receptor, the proposed CB_e_ receptor, the endothelium and nitric oxide (NO). Analysis of AEA intracellular signalling suggests that AEA stimulates eNOS through MAPKs, AKT or Pi3K pathways. Dotted lines show potential linkage of signalling pathways. Fatty acid amide hydrolase (FAAH), cyclooxygenase-1 and -2 (COX1/2), anandamide (AEA), cannabinoid receptor (CB1), putative cannabinoid receptor (CBe), non-specific cannabinoid receptor (CBr), mitogen activated protein kinase family (MAPKs) phosphoinsitide 3-kinase (Pi3K), endothelial derived nitric oxide synthase (eNOS), nitric oxide (NO).

## 1 Introduction

The first discovered endogenous cannabinoid agonist, anandamide (AEA) was shown to induce vasorelaxation of rabbit cerebral arterioles in the early nineties [1]. Since then, AEA is one of the most widely studied cannabinoids in the vasculature [2]. Animal studies have shown that the acute vasorelaxant response to AEA is underpinned by several pathways including cannabinoid (CB_1_, CB_2_ and CB_e_ (proposed cannabinoid receptor located on the endothelium)) receptor activation, activation of transient receptor potential (TRP) channels, with subsequent endothelium derived hyperpolarising factor (EDHF) and/or nitric oxide (NO) mediated relaxation of vascular smooth muscle [3], [4], [5]. The original work of Ellis and colleagues [1] also found that AEA causes vasorelaxation through its metabolism to other vasodilator substances. However, in the rat mesentery, metabolism of AEA appears to limit its vasorelaxant effects [6]. A slowly developing (over 2 h) vasorelaxant response to AEA has also been observed in rat aortae [7], which is inhibited by a peroxisome proliferator-activated receptor gamma (PPARγ) antagonist, endothelium removal, NO synthase and superoxide dismutase inhibition.

Despite the wealth of studies showing that AEA causes acute vasorelaxation of mesenteric arteries in several animal species, the effects of AEA are unknown in human mesenteric arteries. Indeed, investigations into the direct effects of AEA in human vasculature are limited and conflicting. AEA is ineffective as a vasorelaxant in myometrial arteries [8]. However, topical application of AEA causes increased blood flow in the forearm circulation via TRPV1 activation [9]. AEA also causes maximal vasorelaxation of human pulmonary arteries through its metabolism to vasoactive prostanoids and activation of CB_e_ [10]. Interestingly, we recently showed that the mechanisms of action of another endocannabinoid, 2-arachidonoylglycerol (2-AG), is different in human mesenteric arteries compared to that previously observed in animal mesenteric arteries, suggesting further research is required to understand the role and effects of endocannabinoids in human vasculature [11].

Plasma AEA concentrations are reported to be between 0.3–2.5 nmol/L [12], but are raised in patients suffering from diseases that affect the cardiovascular system, for example, in obese patients [13], type-2 diabetics [14], patients with coronary dysfunction [15] and in patients with portal hypertension associated with cirrhosis [16]. Animal studies have shown that increased AEA levels are associated with decreased arterial contractions and enhanced vasorelaxant responses in the mesenteric arteries of biliary cirrhotic rats [17]. Domencali and colleagues [18] also showed that the vasorelaxant response to AEA was enhanced in cirrhotic rats, associated with an increase in CB_1_ and TPRV1 receptor expression. However, in obese rats, anandamide-induced relaxation is decreased in resistance arteries, associated with decreased cannabinoid receptor expression and increased anandamide degradation [19]. We have shown that the responses to AEA are reduced in the Zucker diabetic model, which appears to be brought about by enhanced metabolism of these endocannabinoids, including the production of vasoconstrictor metabolites acting at the thromboxane receptor (Wheal et al., under review). Looking at the effects of patient characteristics on vasorelaxant responses to 2-AG, we found that 2-AG responses were reduced in those with heart disease and type 2 diabetes, and in those taking NSAIDS, statins or anti-diabetic medication [11].

In light of this background, we hypothesised that anandamide would cause acute vasorelaxation of human mesenteric arteries and that these responses would be affected by medical conditions. To address this hypothesis, the aims of the study were to assess the potential vasorelaxant effect of AEA in isolated human mesenteric arteries, to investigate the mechanisms of how this might be brought about, and to establish any potential effect of disease state on AEA responses.

## 2 Methods

### 2.1 Chemicals

All salts, L-NAME, indomethacin and bradykinin were supplied by Sigma Chemical Co. (Poole, UK). AEA, AM251, AM630, and capsaicin were purchased from Tocris (Bristol, UK). L-NAME and indomethacin were dissolved in PSS solution. AEA, bradykinin and capsaicin were all dissolved in ethanol at 10 mmol/L with further dilutions made in distilled water. AM251, O-1918 and AM630 were dissolved in DMSO at 10 mmol/L with further dilutions made in distilled water.

**Fig. 1:**
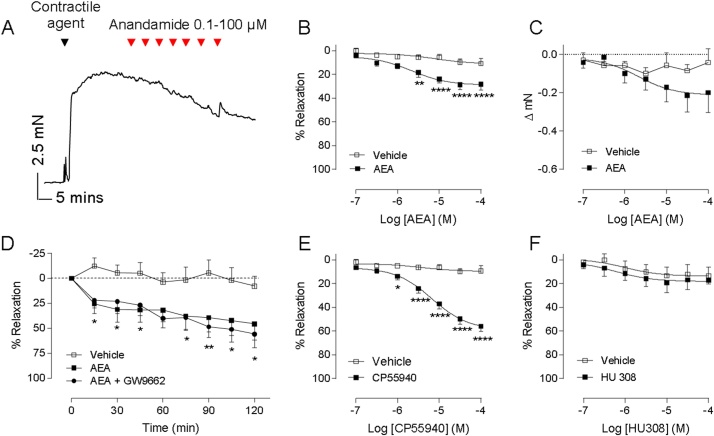
CB_1_ but not CB_2_ agonists cause vasorelaxation of human mesenteric arteries. A representative trace showing a concentration response curve to anandamide (AEA) (A). Graphical representation of concentration-response curves to acute (B, n = 12 patients) and time-dependent (D, n = 5 patients) exposure to anandamide. Concentration-response curves to anandamide in uncontracted arteries (C, n = 7 patients). Concentration responses to the CB_1_ receptor agonist CP55940 (E, n = 12 patients) or the CB_2_ agonist HU308 (F, n = 12 patients). Open squares show data points for EtOH control and closed squares show responses for cannabinoid ligands. Data points represent means with error bars showing SEM. Comparisons between intervention (cannabinoid) and control (vehicle) artery segments from the same patient were made using 2 way repeated measures ANOVA with Sidak’s multiple comparison at each concentration. * *P *< 0.05, ** *P *< 0.01, **** *P *< 0.0001.

Ethical approval was granted by the Derbyshire Research Ethics Committee and Derbyshire Hospitals Trust Research and Development to take mesenteric tissue from 31 patients undergoing surgical treatment of bowel carcinoma and inflammatory bowel disorders. Patient characteristics for those who gave access to medical notes are presented in Table 1. Informed written consent was taken according to the Declaration of Helsinki. Mesenteric tissue containing small mesenteric arteries (700 ± 49 μm diameter (mean ± s.e.m)) were collected, dissected free of all connective tissue and perivascular fat, mounted on to a Mulvany Halpern myograph and normalised to 90% of 13.3 kPa in physiological saline solution (PSS) as previously described [11], [20]. The endothelial response to a single concentration of bradykinin (10 μmol/L) was tested to ensure endothelial integrity, and only vessels showing >70% relaxation were used (mean response was 84 ± 1.6%). After washout, arteries were contracted with a combination of U46619 and endothelin-1. The average level of contraction of all arteries was 17 ± 1 mN (representing 89 ± 4% of the maximal response to a high potassium solution in these arteries). When a stable tone was achieved, cumulative concentration-response curves were constructed to AEA. In acute studies, AEA was added in 5 min intervals, with measurements taken in the final minute of each concentration addition and expressed as percentage relaxation of pre-imposed tone. Vasorelaxant responses were compared to ethanol-treated vehicle controls carried out in adjacent arterial segments from the same patient. In separate, time-dependant studies (see Fig. 1D), a single concentration (10 μmol/L) of AEA was added to pre-contracted arteries of larger diameter (1–2 mm) and changes in tension were recorded for 2 h.

### 2.2 Mechanisms of action

In all interventions to assess mechanisms of action, AEA control responses were carried out simultaneously in adjacent arteries from the same patient. Cannabinoid receptor involvement was assessed with a CB_1_ antagonist AM251 (100 nmol/L, 10 min before contraction of the artery), CB_2_ receptor antagonist AM630 (100 nmol/L, 10 min before contraction of the artery) and CB_e_ antagonist O-1918 (10 μmol/L, 10 min before contraction of the artery). Desensitisation of TRP receptors was achieved via incubation (1 h) with capsaicin (10 μmol/L). The potential involvement of PPARγ was investigated using the antagonist GW9662 (1 μmol/L, 10 min before contraction of the artery). AEA responses were also examined after endothelium denudation (with a human hair), incubation with the fatty acid amide hydrolase (FAAH) inhibitor URB597 (1 μmol/L, 30 min before contraction of the artery and present throughout), the COX inhibitor indomethacin (10 μmol/L, 30 min before contraction of the artery and present throughout) or the NOS inhibitor, Nω-Nitro-l-arginine methyl ester hydrochloride (L NAME, 300 μmol/L, present throughout). None of the interventions tested significantly affected the levels of tone prior to addition of AEA.

### 2.3 Cell culture

Human aortic endothelial cells (HAECs, PromoCell, Germany, passage 4) were grown in PromoCell Endothelial Cell Growth medium to confluence on 6 well plates and treated for 10 min with 10 μmol/L AEA or the synthetic CB_1_ agonist CP55950 (1 μmol/L), after which time the medium was removed and the cells collected in cell lysis buffer (RIPA buffer, SigmaAldrich) with phosphatase and protease inhibitors (Roche). The protein concentration of the cell lysate was measured using a BCA assay (Sigma Aldrich). The levels of phosphorylated ERK/MAP kinase 1/2 (Thr185/Tyr187), Akt (Ser473), STAT3 (Ser727), JNK (Thr183/Tyr185), p70 S6 kinase (Thr412), NFκB (Ser536), STAT5A/B (Tyr694/699), CREB (Ser133), and p38 (Thr180/Tyr182) were measured in cell lysates using the Luminex^®^ xMAP^®^ technology using a commercially available panel for screening signalling pathways (Milliplex™, 48-680MAG, Merck Millipore), and normalised to total protein content.

### 2.4 RT-PCR

The presence of predicted sites of action was investigated at the mRNA level using reverse transcription followed by polymerase chain reaction (RT-PCR) as previously published [20], [21]. Human astrocytes (HAs) were used as a positive control known to express all the target sites of action of interest [21]. Total RNA was extracted from HAs and HAECs using Allprep DNA/RNA kit with on column DNaseI treatment (Qiagen, Germany). Reverse transcription with (+) and without reverse transcriptase (−) was performed in 20 μl final volume using 2 μg of total RNA and random primers with the High Capacity cDNA Reverse Transcription Kit (Life Technologies, UK). PCR reactions were carried out in a final volume of 25 μl with Zymotaq (ZymoResearch, USA) using 2 μl of reverse transcription product as the template. After 5 min at 95 °C, PCRs were performed for 40 cycles except those for CGRPR and CB2R that were carried out for 60 cycles. The cycles included 30 s at 95 °C, 30 s at the annealing temperature that was optimal for each primer pair (56 °C for CB1R and CB2R; 60 °C for HPRT; 58 °C for TRPV1; 61 °C for CGRPR) and a final extension step of 30 s at 72 °C. Amplification products were separated by gel electrophoresis through ethidium bromide stained 2% agarose (CB1R, CB2R, TRPV1, CGRPR and HPRT) and visualised using a Biorad Chemidoc.

### 2.5 Statistical analysis

Graphs represent mean percentage relaxations, with error bars representing the standard error of the mean (SEM), and n equalling the number of patients. R_max_ and EC_50_ values were obtained from sigmoidal concentration-responses curves (Prism Version 5; GraphPad Software, California, USA). Comparisons between intervention and control artery segments from the same patient were made using 2 way repeated measures ANOVA with Sidak’s multiple comparison at each concentration. The area under the curve (AUC) of concentration-response curves was calculated using Prism and this data was used to identify any statistical outliers using the Rout method. One patient was excluded from the final analysis based on this outlier analysis. The potential relationship between patient characteristics and the anandamide AUC responses (which were normally distributed) were analysed by Pearsons correlation. Significance was determined at P < 0.05.

## 3 Results

### 3.1 AEA causes acute and time-dependent vasorelaxation of human mesenteric arteries

AEA caused acute vasorelaxation of human mesenteric arteries (700 ± 49 μm diameter) with an R_max_ 29 ± 3% and EC_50_ −5.7 ± 0.3 significantly different to vehicle between 3 and 100 μmol/L (n = 12, Fig. 1A & B). AEA caused a modest (but not significant) reduction in baseline tone when added to uncontracted arteries (Fig. 1C). As AEA is a partial agonist of both CB_1_ and CB_2_ receptors, full agonists of these receptors, CP55,940 (CB_1_) and HU308 (CB_2_), were also tested. CP55,940 caused vasorelaxation with an R_max_ 60 ± 3.6% and EC_50_ −5.2 ± 0.1 significantly different to vehicle control from 1 μmol/L (n = 12, Fig. 1E). HU308 had no effect on vascular tone compared to vehicle (Fig. 1F).

When a single concentration of AEA (10 μmol/L) was added in arteries of a larger diameter (1–2 mm), AEA caused an initial vasorelaxation that was significantly different to vehicle control (P < 0.05), non-recoverable and increased over time (see Fig. 1D). This was not affected by the PPARγ antagonist GW9962 (Fig. 1D).

### 3.2 AEA-induced vasorelaxation is not limited by its metabolism, but is endothelium-dependent

**Fig. 2:**
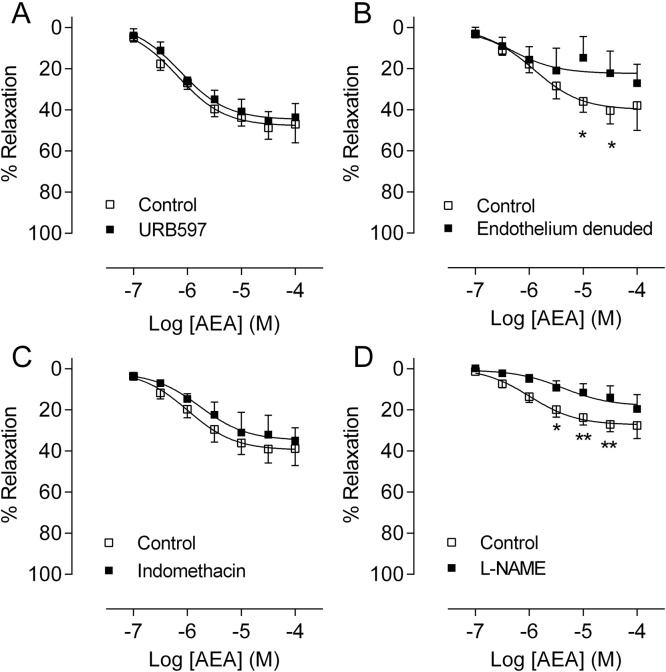
Anandamide-induced vasorelaxation is not altered by inhibition of its metabolism, but it is endothelium-dependent. Anandamide concentration-response curves in the presence of the fatty acid amide hydrolase inhibitor URB597 (A, n = 5 patients), after removal of the endothelium (B, n = 5 patients), in the presence of the non-selective cyclooxygenase inhibitor indomethacin (C, n = 7 patients), and in the presence of the nitric oxide synthase inhibitor L-NAME (D, n = 6 patients). Open squares show data points for anandamide control and closed squares show responses for anandamide with intervention. Data points represent means with error bars showing SEM. Comparisons between intervention and control (AEA alone) artery segments from the same patient were made using 2 way repeated measures ANOVA with Sidak’s multiple comparison at each concentration. * *P *< 0.05, ** *P *< 0.01.

Incubation with the FAAH inhibitor URB597 (Fig. 2A) or the COX inhibitor indomethacin or (Fig. 2C) did not modify AEA concentration‐response curves, therefore, no further experiments were performed to test prostanoids (or other metabolite) pathways. Removal of the endothelium inhibited the vasorelaxant effects of AEA (P < 0.05, Fig. 2B). Incubation with L-NAME also reduced the vasorelaxant effects of AEA (P < 0.01–0.05, Fig. 2D).

### 3.3 AEA-induced vasorelaxation is dependent on CB_1_ and CB_e_ but not CB_2_ or TRPV channels

**Fig. 3:**
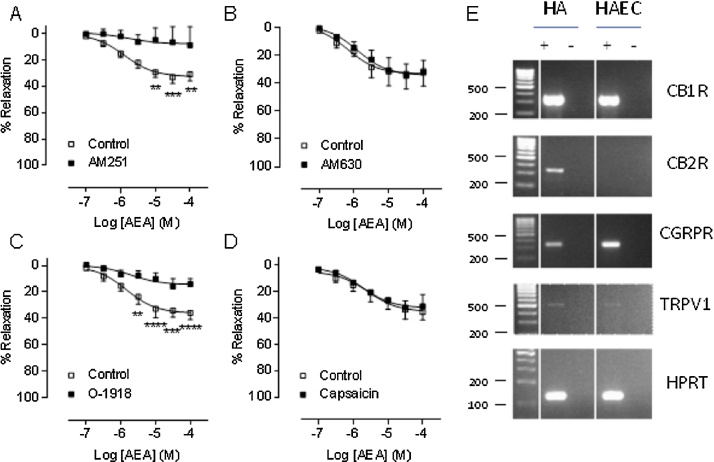
Anandamide-induced vasorelaxation is reduced by antagonism of the CB_1_ and CB_e_ receptors. Anandamide (AEA) concentration-response curves in the presence of the CB_1_ inhibitor AM251 (A, n = 6 patients), the CB_2_ inhibitor AM630 (B, n = 4 patients), the CB_e_ inhibitor O-1918 (C, n = 5 patients), and after desensitisation of the TRPV receptors using capsaicin (D, n = 4 patients). Open squares show data points for AEA control responses and closed squares show responses for AEA with intervention. Data points represent means with error bars showing SEM. Comparisons between intervention and control (AEA alone) artery segments from the same patient were made using 2 way repeated measures ANOVA with Sidak’s multiple comparison at each concentration. ** *P *< 0.01, *** *P *< 0.001, **** *P *< 0.0001. E. RT-PCR showing the presence of CB_1_, TRPV1, and CGRP receptors, but not CB_2_, in human aortic endothelial cells (HAECs). Hypoxanthine-guanine phosphoribosyltransferase (HPRT) was used as a house-keeping gene. The columns shown with + are with reverse transcriptase and those with − are without reverse transcriptase. Human astrocytes (HA) are shown as a positive control for CB_2_ expression.

Antagonism of the CB_1_ receptor using AM251 (100 nmol/L) inhibited AEA responses from 3 μmol/L (Fig. 3A). Antagonism of the CB_2_ receptor (AM630, 100 nmol/L) did not alter AEA‐induced relaxation (Fig. 3B), consistent with the lack of a vasorelaxant response to the CB_2_ agonist HU308 (Fig. 1F). Antagonism of the putative CB_e_ receptor using O-1918 (1 μmol/L), inhibited AEA responses from 3 μmol/L (Fig. 3C). Desensitisation of TRPV receptors using capsaicin (10 μmol/L) did not affect AEA-induced vasorelaxation (Fig. 3D). To confirm the presence of these target sites at the RNA level in the endothelium (see Fig. 2B), RT-PCR was carried out in human aortic endothelial cells (HAECs) where the expression of presence of CB_1_ but not CB_2_ receptors was shown using relevant primers (Fig. 3E). As a positive control, the expression of CB_2_ was observed in human astrocytes [21] (Fig. 3E). Although AEA responses were not affected by capsaicin pre-treatment (Fig. 3D) as in animal studies [22], TRPV1 and the CGRP receptor mRNA were present in HAECs.

### 3.3 Intracellular signalling responses to AEA and CP55940 in HAEC cells

**Fig. 4:**
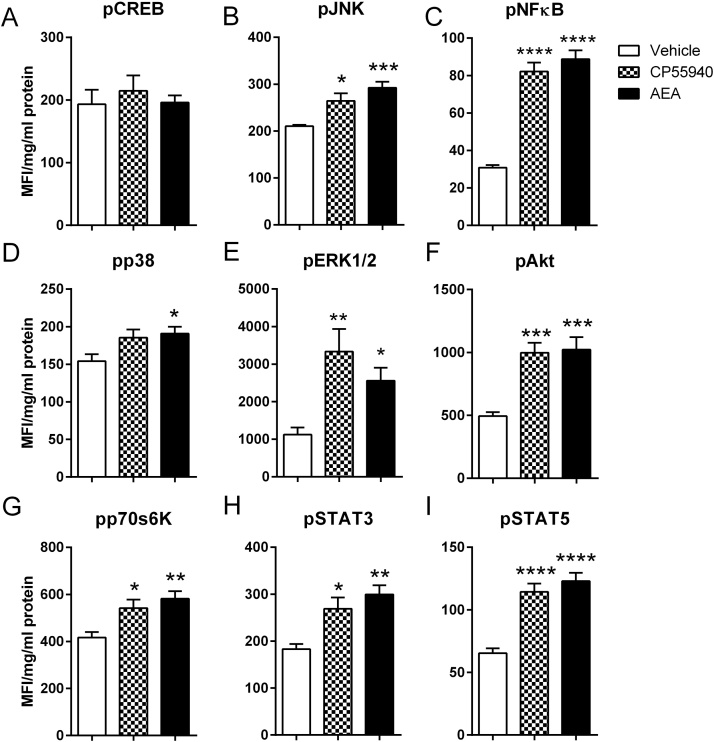
Anandamide and CP55940 show similar profiles of intracellular signalling proteins in human aortic endothelial cells. Levels of phosphorylated CREB (A), JNK (B), NFκB (C), p38 (D), ERK/MAP kinase 1/2 (E), Akt (F), p70s6K (G), STAT3 (H) and STAT5 (I) were measured in endothelial cell lysates after 10 min incubation with anandamide (AEA; 10 μM) or CP55940 (1 μM) using the Luminex^®^ xMAP^®^ technology and normalised to total protein content. Data are presented as mean with SEM and were analysed by one-way ANOVA with Dunnett’s comparison against the vehicle control response (n = 6). ** *P *< 0.01, *** *P *< 0.001, **** *P *< 0.0001.

In HAECs, AEA (10 μmol/L) and the CB_1_ agonist CP55,940 (1 μmol/L) significantly increased the levels of phosphorylated JNK, NFκB, p70s6K, STAT3, STAT5, ERK 1/2 and Akt to similar levels (Fig. 4B– I). AEA, but not CP55,940, also increased phosphorylation of p38 (Fig. 4D). There was no significant difference between AEA and CP55,940 in any of the signalling proteins tested.

### 3.4 The effects of patient characteristics on AEA responses

**Fig. 5:**
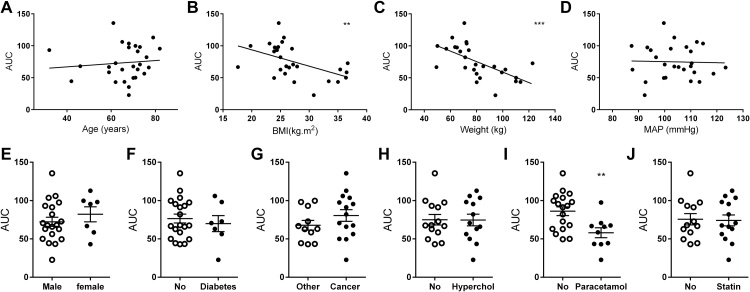
Post-hoc analysis of anandamide responses. Correlation between the total response to AEA (area under the curve, AUC) and age (A), BMI (B), body weight (C) and mean arterial pressure (D). AUC responses to AEA in males and females (E), patients with and without type 2 diabetes (F), cancer (G), hypercholesterolaemia (H) or those taking paracetamol (I) or statins (J). A–D were analysed by Pearsons correlation and E–J were analysed by unpaired *t*-test. **P < 0.01, ***P < 0.001.

Considerable differences were observed in the AEA vasorelaxant responses across patients. Therefore, post-hoc analysis of patient responses was carried out to establish any potential relationships between AEA-induced vasorelaxant and patient characteristics. Vasorelaxant responses to AEA did not correlate with patient age (r = 0.1025, Fig. 5A), gender (Fig. 5E) or mean arterial blood pressure (r = −0.0292, Fig. 5D; SBP and DBP were also not correlated with AEA responses, data not shown). A significant negative correlation was observed between the response to AEA (total response to AEA calculated as the area under the curve (AUC)) to BMI (r = −0.5003, P < 0.01, Fig. 5B) and body weight (r = −0.6096, P < 0.0001, Fig. 5C). AEA responses were not altered in patients with diabetes (Fig. 5F), cancer (Fig. 5G), or hypercholesterolaemia (Fig. 5H) or in patients taking ACE inhibitors, hypoglycaemic agents, NSAIDS, beta-blockers (data not shown) or statins (Fig. 5J). However, the vasorelaxant response to AEA was significantly blunted in those taking paracetamol/co-codamol (P < 0.01, Fig. 5I).

## 4 Discussion

The aim of this study was to explore the effects of AEA in human mesenteric arteries, to establish the target sites at which AEA acts, and test if the effects of AEA were affected by patient characteristics. AEA caused moderate endothelium-dependent vasorelaxation of human mesenteric arteries, which was inhibited by antagonism of CB_1_ and an uncharacterised endothelial cannabinoid receptor (CB_e_). CB_2_ activation did not cause vasorelaxation of human mesenteric arteries. The mechanisms underpinning vasorelaxation to AEA show both similarities (roles for the endothelium and CB_1_) and differences (no roles for metabolism or TRP channel activation) to that observed in animal studies. AEA-induced vasorelaxation was not significantly altered by gender, age or patient medical conditions, but was reduced in overweight patients, and in those taking paracetamol containing medications.

Previous studies in rat mesenteric arteries have shown that low micromolar concentrations of AEA cause maximal vasorelaxation [5], [6]. In human pulmonary arteries, AEA also caused maximal vasorelaxation [23]. However, in the present study, both the potency and efficacy of AEA were lower. As metabolism of AEA has been shown to limit its vasorelaxant effects in the rat mesentery [6], we hypothesised that the lower efficacy of AEA might be because of its degradation in human mesenteric arteries. However, we found that inhibition of FAAH or COX had no effect on the vasorelaxant response to AEA. This is interesting because we recently found that metabolism of 2-AG to vasoactive substances was the main mechanism of action underpinning vasorelaxation to 2-AG in human mesenteric arteries [11]. This could be because 2-AG is less metabolically stable than AEA, and may explain the reduced efficacy of AEA in the present study compared to 2-AG [11]. Another possible explanation for the reduced efficacy of AEA is that our patient population were undergoing surgery, were older and had a variety of comorbidities potentially associated with vascular dysfunction. Indeed our post hoc analysis showed that AEA responses were blunted in overweight patients and those taking paracetamol. However, when we looked at the responses curves to AEA in the patients with a BMI less than 25 and not taking paracetamol, the maximal vasorelaxant response to AEA was still only 44% (n = 6), suggesting a lower maximal response to AEA in human mesenteric arteries is likely.

In line with many animal studies [2], we found that the vasorelaxant response to AEA was inhibited by CB_1_ receptor antagonism. We also found that a synthetic CB_1_ agonist (CP55,940) caused vasorelaxant in human mesenteric arteries (albeit a greater R_max_, probably reflecting the greater potency and efficacy at CB_1_), and confirmed the presence of CB_1_ mRNA in human aortic endothelial cells (which we have also demonstrated in human brain microvascular endothelial cells [21]). In these cells, AEA shared a similar signalling profile as the archetypical CB_1_ ligand CP55,940, and caused phosphorylation of the intracellular proteins p38 MAPK, ERK1/2 MAPK and Akt, all of which have previously been associated with AEA signalling [39], [40] and activation of eNOS [41]. Taken together with the L-NAME data, this suggests that part of the vasorelaxation seen to AEA in human arteries is through cannabinoid receptor signalling to MAPKs and Akt activating eNOS. Other human studies, albeit in pulmonary arteries, have not found a role for the CB_1_ receptor in cannabinoid-induced vasorelaxation [10]. This probably reflects regional difference in vascular CB_1_ expression.

In contrast, the CB_2_ ligand HU-308 had no effect on vascular tone, and the vasorelaxant response to AEA was not affected by CB_2_ receptor antagonism. This is in agreement with a lack of a role for CB_2_ in cannabinoid-mediated vasorelaxation in animal studies [2]. We also found that CB_2_ was not expressed in human aortic endothelial cells. It is likely, however, that CB_2_ is upregulated in the vasculature in pathologies such as atherosclerosis, where an anti-inflammatory role for CB_2_ activation has been identified [25], and we recently showed that high glucose or high insulin treatment increases the mRNA of CB_2_ in endothelial cells [20].

The existence of a novel cannabinoid receptor located exclusively on the endothelium was first suggested in 1999 [26]. This receptor has been implicated in animal studies to be involved in mediating vasorelaxation to a range of cannabinoids in a variety of vascular beds, and is antagonised by an analogue of cannabidiol, O1918 [5], [26], [27], [28]. In agreement with these studies, we found that the effects of AEA in human mesenteric arteries were reduced by removal of the endothelium and in the presence of O-1918, further suggesting the existence of the proposed CB_e_ receptor in humans [10], [23], [29]. The exact identity of this receptor is still unknown but a promising candidate is the orphan receptor GPR18. It follows a similar ligand binding profile to that of the CB_e_ receptor [30], and has been shown to be present in the endothelium of rat retinal arteries [31].

The response of AEA was unaltered by TRPV channel desensitisation by capsaicin in the present study. This is in contrast to animal studies, which have shown a significant role of TRPV activation with subsequent CGRP release [2]. We have recently shown a role for the TRPV1 channel in mediating the vasorelaxant response to CBD [32] and capsaicin (unpublished data) in the same human mesenteric arteries, and have demonstrated the presence of both TRPV1 and the CGRP receptor in human aortic endothelial cells. Thus, the issue is unlikely to be that TRPV1 is not expressed in these arteries. AEA requires facilitated transport across the cell membrane to activate TRPV1 [33], therefore a possible explanation is that insufficient levels of AEA are reaching the intracellular binding site of TRPV1.

In larger conduit arteries such as the aorta and superior mesenteric artery, we have shown that cannabinoids, including AEA, cause a time-dependent vasorelaxation mediated by PPARγ [34], [35]. To test whether a similar response to AEA was observed in human mesenteric arteries, we selected arteries of the largest diameter available to us in the mesenteric samples (1–2 mm). Application of a single concentration AEA caused a non-recoverable vasorelaxation significantly different to vehicle over 2 h. However, unlike in the rat aorta, this response was not affected by a PPARγ antagonist. This is probably due to the size of the arteries in the present study, as we previously showed that the PPARγ-mediated time-dependent response to AEA was only observed in larger conduit arteries such as the aorta and superior mesenteric artery, and not in smaller resistance vessels of the mesenteric bed [36].

To identify any trends in the effects of patient characteristics, co-morbidities or medication on AEA responses, we carried out post hoc analysis which revealed two influences on AEA responses; BMI/body mass and paracetamol. A similar effect of obesity on AEA response has been identified in Zucker rats [19], where the reduced vasorelaxation to AEA was due to decreased CB_1_ and TRPV1 contribution, increased AEA degradation, and a decreased ability of AEA to stimulate eNOS. There is also evidence in the literature of interactions between the endocannabinoid system and paracetamol. AM404 is a metabolite of paracetamol that inhibits AEA transport and FAAH activity which would prevent AEA degradation, [37] and AM404 also activates TRPV1[38]. Thus taking paracetamol may interfere with the vascular actions of AEA, which warrants further investigation.

In conclusion, AEA causes endothelium-dependent, NO-dependent vasorelaxation in human mesenteric arteries that is less efficacious than in animal models. Unlike in rat mesenteric arteries, AEA‐induced vasorelaxation in human mesenteric arties is not limited by its metabolism or inhibited by TRPV channel desensitisation. However, similar to that observed in rat mesenteric arteries, AEA-mediated vasorelaxation is dependent on CB_1_ and CB_e_ receptor activation. Post hoc analysis of the data set suggest that obesity and taking paracetamol reduce the vasorelaxant response to AEA. These findings demonstrate a role for AEA in modulating vascular tone in humans mediated by CB_1_ and the endothelium.

## Funding

This work was supported by the British Heart Foundation (FS/09/061)

## Disclosures

All authors declare no conflict of interest.

## Author contribution

C P Stanley, W H Hind, C Tufarelli and S E O’sullivan performed the research and approved the final version.

C P Stanley and S E O’sullivan designed the research study and approved the final version.

C P Stanley and S E O’sullivan analysed the data and approved the final version.

C P Stanley and S E O’sullivan wrote the paper and approved the final version.

## Figures and Tables

**Table 1:** Patient characteristics.

Characteristic	Range	Mean ± SEM
Ethnicity	27 UK white	
Male	21	
Female	6	
Age	32–82	66 ± 2
Weight (kg)	49–122	81 ± 4
BMI (kg/m^2^)	17.6–36.7	27.4 ± 1
Smoking habits		
Non smokers	21	
0–10 CPD	3	
10–20 CPD	3	
Drinking habits		
<10 units p/w	17	
10–20 units p/w	8	
>20 units p/w	2	
Operation		
Right Hemicolectomy	7	
Left Hemicolectomy	2	
Sigmoid Colectomy	7	
Anterior Resection	6	
Abdominoperineal Resection	1	
Total colectomy	4	
Reason for surgery		
Cancer	16	
Inflammatory bowel disorder	11	
Dukes Staging		
Dukes A	8	
Dukes B	4	
Dukes C	3	
Dukes D	1	
Systolic Blood Pressure (mm/Hg)	110–172	142 ± 3
Diastolic Blood Pressure (mm/Hg)	65 ± 101	84 ± 2
Diabetic	7	
Heart Disease	22	
Heart Failure	0	
Hypercholesterolemia	13	
Hypertensive	15	
α-1 adrenoceptor antagonist (total)	1	
Alfuzosin	0	
terazocin	1	
ACE Inhibitors (total)	7	
Lisinopril	5	
Ramipril	2	
AT1 receptor antagonists (total)	2	
Losartan	1	
Irbesartan	1	
Beta Blockers (total)	5	
Metoprolol	1	
Atenolol	3	
Propranolol	1	
Calcium channel blocker (total)	2	
Amlodipine	1	
Nifedipine	1	
Lodipine	0	
Digoxin	1	
Diuretics (total)	2	
Furosemide	2	
GTN	3	
Hypoglycaemic Medication (total)	5	
Gliclazide	5	
Metformin	4	
Analgesia Medication (total)	12	
Aspirin	4	
Ibuprofen	1	
Paracetamol	6	
CoCodamol	3	
Tramadol	2	
Statin (total)	14	
Atorvastatin	4	
Simvastatin	9	
Pravastatin	1	
Thiazolidinedione (total)	1	
Pioglitazone	1	
